# Improved T Cell Surfaceomics by Depleting Intracellularly Labelled Dead Cells

**DOI:** 10.1016/j.mcpro.2025.101503

**Published:** 2025-12-24

**Authors:** Christofer Daniel Sánchez, Aswath Balakrishnan, Blake Krisko, Bulbul Ahmmed, Luna Witchey, Oceani Valenzuela, Minas Minasyan, Anthony Pak, Haik Mkhikian

**Affiliations:** Department of Pathology and Laboratory Medicine, University of California, Irvine, California, USA

**Keywords:** biotinylation, glycosylation, intracellular pool, plasma membrane, surfaceome

## Abstract

Although the plasma membrane (PM) is among the most biologically important and therapeutically targeted cellular compartments, it is among the most challenging to faithfully capture using proteomic approaches. The quality of quantitative surfaceomics data depends heavily on the effectiveness of the cell surface enrichment used during sample preparation. Enrichment improves sensitivity for low abundance PM proteins and ensures that the changes detected reflect PM expression changes rather than whole cell changes. Cell surface biotinylation with PM-impermeable, amine-reactive reagents is a facile, accessible, and unbiased approach to enrich PM proteins. However, it results in unexpectedly high contamination with intracellular proteins, reducing its utility. We report that biotinylating human cells with amine-reactive reagents intracellularly labels a small but reproducible population of nonviable cells. Although these dead cells represent only 5 ± 2% of the total, we find that in T cell preparations the dead cells account for 90% of labelled proteins. Depleting Annexin V positive dead T cells postlabelling removes ∼99% of the intracellularly labelled cells, resulting in markedly improved PM identifications, peptide counts, and intensity-based absolute quantification intensities. Correspondingly, we found substantial depletion of intracellular proteins, particularly of nuclear origin. Overall, the cumulative intensity of PM proteins increased from 4% to 55.8% with dead cell depletion. Finally, we demonstrate that immature ER/Golgi glycoforms of CD11a and CD18 are selectively removed by dead-cell depletion. We conclude that high intracellular labelling of nonviable cells is the major source of intracellular protein contaminants in amine-reactive surface enrichment methods and can be reduced by dead-cell depletion postlabelling, improving both the sensitivity and accuracy of PM proteomics.

## Highlights

• Amine-labelling based surfaceomics suffers from high intracellular contamination.
• Non-viable cells are the major source of intracellular contaminants in Jurkat cells.
• Dead cell depletion *after* biotinylation removes ∼99% of dead cells.
• Cell surface protein intensity increased >10-fold with depletion.
• Intracellular pools of PM proteins are removed but not surface-resident proteins.

## Graphical Abstract

**Figure:**
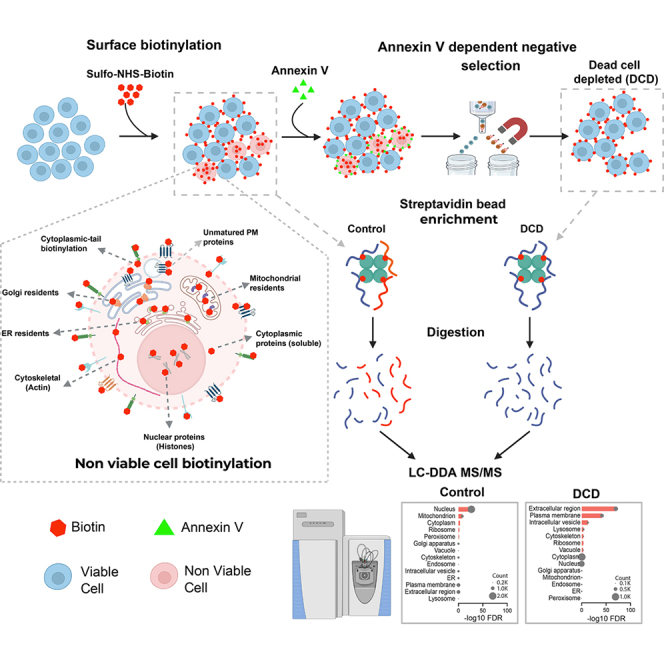

## In Brief

Surfaceomics reliant on amine-reactive biotinylation suffers from high contamination due to non-viable cells that are intensely intracellularly labeled. Even at low frequencies, these cells disproportionately contaminate surfaceomics studies with highly abundant intracellular proteins. Depletion of Annexin V-positive dead cells after labelling removes ∼99% of these biotin^high^ cells, markedly improving plasma membrane (PM) protein enrichment and sensitivity. This method increased cumulative PM protein intensity from 4% to 55.8% and selectively removed intracellular pools of PM proteins while retaining surface-resident pools.

The cell surface proteome, or surfaceome, serves as a critical interface between a cell and its environment, orchestrating essential functions such as signal transduction, nutrient uptake, and immune recognition (1). Consistent with its biological importance, 50% of small molecule drugs and virtually all immunotherapies target plasma membrane (PM) proteins (2, 3). Most PM proteins are transmembrane glycoproteins synthesized in the endoplasmic reticulum (ER) (4). They traffic through the secretory pathway where their glycans are variably processed, delivering a heterogenous set of glycoforms to the PM (5). After variable PM retention times, these glycoproteins are internalized and further trafficked through the endosomal system, frequently being recycled to the cell surface before finally being degraded in lysosomes (6).

Given the biological importance and therapeutic relevance of the PM compartment, there is a need for techniques that allow accurate and sensitive profiling of the surfaceome. Mass spectrometry-based proteomics approaches are powerful in this regard but must be combined with PM enrichment. Enrichment is required to both increase sensitivity for low abundance PM proteins, which collectively constitute ∼1% of the cellular proteome, and to distinguish cell surface from intracellular pools of PM proteins (7). Several strategies have been developed to enrich PM proteins, including density gradient fractionation, and chemical and enzymatic labelling methods coupled to affinity purification (8). The most prominent direct chemical labelling approaches target amines (lysines) or aldehydes (glycans) with membrane impermeable biotinylation reagents such as sulfo-NHS-biotin (9).

Glycan labelling results in fewer intracellular contaminants than amine labelling, but relies on the presence of glycans. Though unglycosylated PM proteins are in the minority, they escape capture by glycan labelling. Furthermore, sialylated glycans are more readily labelled by these reagents (10). Thus, quantitative comparisons, and particularly those that involve conditions that may perturb the glycans themselves would suffer from glycoform bias. Amine-labelling avoids these issues by targeting the aglycone component of glycoproteins. However, it suffers from markedly more contamination by intracellular proteins (9, 10). These contaminants reduce detection of low abundance PM proteins and obscure true PM signal by preventing discrimination between surface-localized and intracellular pools of the same protein. This is particularly problematic for heavily trafficked or recycled proteins, but is a common feature of all PM proteins since they are synthesized intracellularly (11).

The source of intracellular protein contamination in amine-labelling studies is incompletely understood and is the focus of this study. Using amine-reactive reagents we demonstrate that biotinylation results in a small but reproducible population of intracellularly labelled cells which disproportionately contaminate downstream affinity purification of PM proteins. We further demonstrate that postlabelling depletion of Annexin V positive dead cells removes ∼99% of intracellularly labelled cells and markedly improved PM identification and peptide coverage. Moreover, using lymphocyte function-associated antigen 1 (LFA-1) as an example we show that dead cell depletion selectively reduces the intracellular pool of PM proteins. Overall, we identify a major source of contamination in amine-labelling PM enrichment, establish a simple and highly effective approach to remove these contaminants, and show that this increases both the sensitivity and accuracy of surfaceomics.

## Experimental Procedures

### Experimental Design and Statistical Rationale

This study aimed to identify the source of intracellular protein contaminants in plasma membrane (PM) enrichment proteomics from cultured cells. To investigate the origin of nonspecific intracellular contaminants, we employed a flow cytometry-based approach to evaluate multiple amine-reactive biotinylation reagents. These experiments revealed that compromised cells present during surface labeling—specifically dead or dying cells-are a primary source of intracellular contamination. To address this, we systematically compared several Annexin V-based depletion strategies to selectively remove nonviable cells prior to biotinylation. PM protein enrichment efficiency was assessed using two independent biological replicates of surface biotinylation and streptavidin-based affinity purification in Jurkat cells, followed by data-dependent acquisition mass spectrometry on a Thermo Orbitrap Fusion Lumos. Raw data were processed with MaxQuant v2.1.3.0 using the human UniProt database, with a 1% false discovery rate (FDR) applied at both the peptide and protein levels (12). The mass spectrometry data displayed in the main figures is from a representative replicate selected for optimal streptavidin pull-down efficiency and protein yield; a second independent replicate was performed, showed consistent plasma membrane protein enrichment, and is presented in the supplement. Although selected figures present single-replicate results, the reproducibility of labeling patterns across biological replicates supports the robustness and reproducibility of the findings. Statistical comparisons between experimental conditions were performed using unpaired two-tailed t-tests with Welch’s correction to account for unequal variances.

**Fig. 1:**
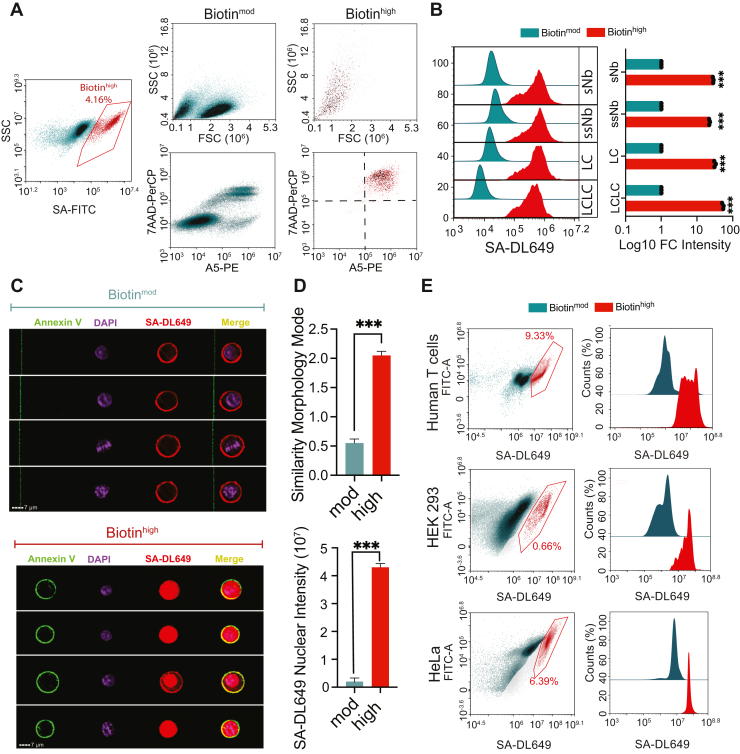
**NonViable Cells Drive Intracellular Protein Contamination in Amine-Reactive Surface Biotinylation**. *A*, jurkat cells were labeled with sulfo-NHS-biotin and stained with streptavidin-FITC, 7-AAD-PerCP, and Annexin V-PE to assess biotinylation levels and cell viability by flow cytometry. Dot plots gated on biotin^high^ (*right*) and biotin^mod^ (*middle*) are shown. *B*, jurkat cells were biotinylated using one of four NHS-biotin variants: sulfo-NHS-biotin (sNb), sulfo-NHS-SS-biotin (ssNb), sulfo-NHS-LC-biotin (LC), or sulfo-NHS-LCLC-biotin (LCLC). Cells were then stained with streptavidin-AF647, and mean fluorescence intensities (MFI) of biotin^mod^ and biotin^high^ populations were quantified by flow cytometry. *C*, Representative images of Jurkat cells labeled with sulfo-NHS-biotin, stained with streptavidin-AF647, Annexin V, and DAPI, and acquired using the ImageStreamX Mark II imaging flow cytometer to visualize biotin localization and cell viability. (*D*) Cells were gated based on biotin signal intensity, and nuclear colocalization of the biotin signal (*top*) and nuclear streptavidin staining intensity (*bottom*) were quantified using IDEAS software (https://cytekbio.com/pages/imagestream). *E*, human T cells, HeLa, and HEK293 cells were biotinylated with sulfo-NHS-biotin, stained with streptavidin-AF647, and analyzed by flow cytometry to examine biotin^high^ populations across cell types. ∗∗∗*p* < 0.001, ∗∗∗∗*p*-value <0.0001 by unpaired two-tailed *t* test with Welch’s correction.

**Fig. 2:**
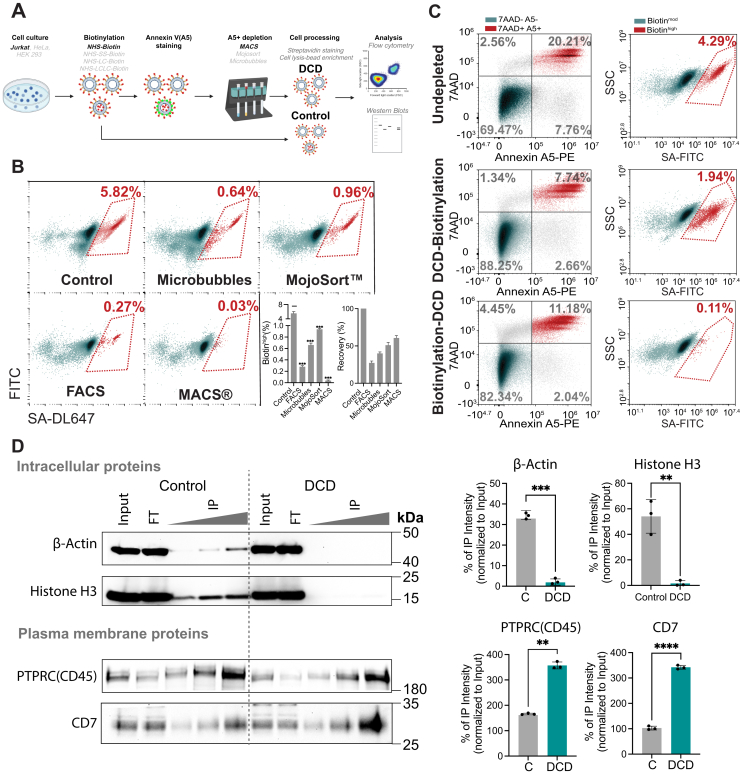
**Optimization of Dead Cell Removal Strategies to Minimize Intracellular Protein Contamination Following Amine-Reactive Biotinylation**. *A*, schematic illustration of the experimental workflow, depicting NHS-biotin surface labeling followed by dead cell depletion. *B*, Annexin V–positive cells remaining after NHS-biotin surface labeling were depleted using various methods as indicated. The efficiency of depletion and cell recovery rates were quantified by flow cytometry. *C*, jurkat cells containing Annexin V–positive cells were either *left* undepleted (*top panel*), depleted prior to surface labeling (*middle panel*), or depleted after surface labeling (*bottom panel*). Cells were stained with streptavidin to assess the efficiency of removing the Biotin^high^ population by flow cytometry. *D*, surface-labeled cells, with or without Annexin V–positive cell removal, was lysed, and biotinylated proteins were enriched using streptavidin-coated agarose beads at 4 °C overnight. Postenrichment, proteins were eluted and loaded onto SDS-PAGE gels at varying volumes (10 μl, 20 μl, and 40 μl), alongside input and flow-through fractions. Western blotting was performed using primary antibodies against Actin, Histone H3, PTPRC (CD45), and CD7, followed by HRP-conjugated secondary antibodies. Band intensities were quantified using ImageJ, and immunoprecipitated signals were normalized to input to calculate the percentage of enrichment. NS, not significant; ∗*p* < 0.05; ∗∗*p* < 0.01; ∗∗∗*p* < 0.001 by unpaired two-tailed *t* test with Welch’s correction.

**Fig. 3:**
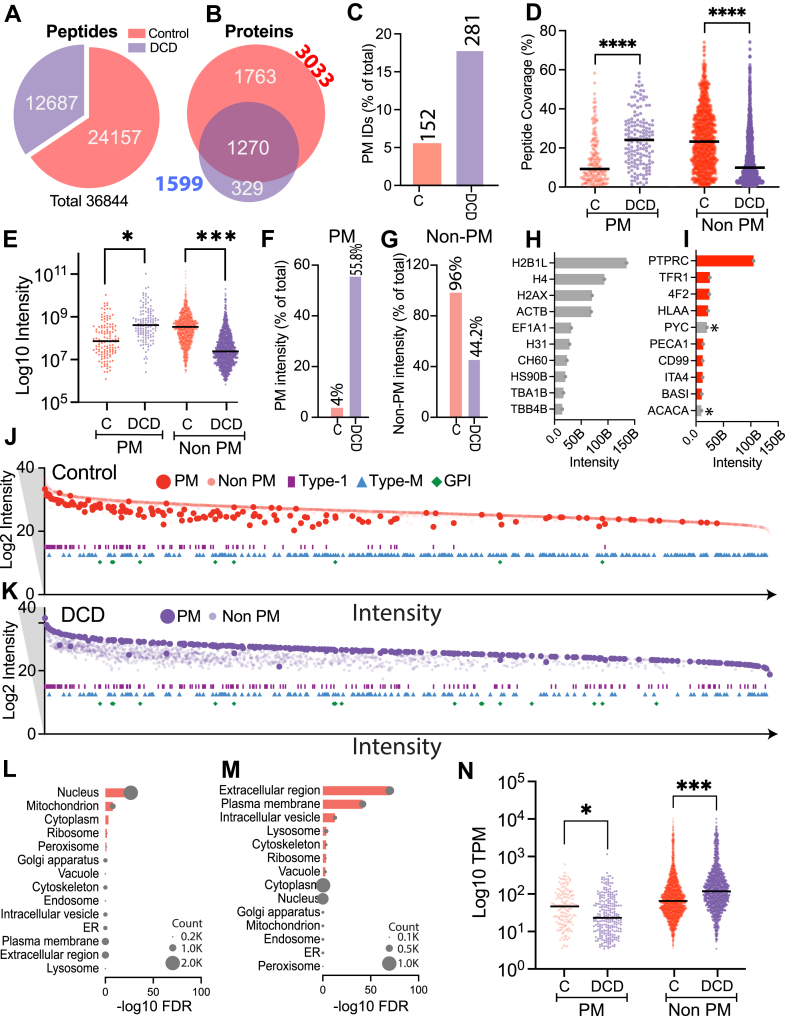
**Annexin V–Based Dead Cell Depletion Enhances Specificity and Sensitivity of Plasma Membrane Proteomics by Mass Spectrometry**. Peptide (*A*) and protein (*B*) overlaps between control and dead cell depletion (DCD) runs are shown. *C*, quantification of plasma membrane (PM) proteins in control and DCD samples was performed based on SURFY annotations. Peptide coverage percentages for PM and non-PM (*D*) and iBAQ intensities (*E*) are compared between control and DCD samples in (*D*). iBAQ intensities. The percentage intensity of PM proteins (*F*) and Non-PM proteins (*G*) was calculated relative to the total protein intensity within each sample. Comparisons were made between control and DCD groups to assess distribution differences. *Bar plots* illustrate the top 10 highest-abundance proteins based on raw intensity in control (*H*) and DCD (*I*) samples, with *red bars* indicating SURFY-annotated PM proteins and *asterisks* denoting endogenous biotinylated proteins. *J* and *K*, scatter plots depict proteins identified in control (*J*) and DCD (*K*) datasets, where PM proteins are shown as *large*, *dark circles* (*red* in control, *blue* in DCD) and non-PM proteins as *smaller*, *lighter circles*. Protein features are annotated as *purple rectangles* for type I membrane proteins, *cyan triangles* for multi-pass proteins, and green diamonds for GPI-anchored proteins. The x-axis represents summed intensities across control and DCD datasets, and the y-axis indicates intensities in either control or DCD samples. *L* and *M*, subcellular enrichment analyses were performed using SubcellulaRVis with the Jurkat genome as background, with *red bars* indicating significantly enriched compartments and bubble sizes reflecting the number of proteins identified in control (*L*) and DCD (*M*) samples. *N*, comparison of Human Protein Atlas–derived expression levels is shown for PM and non-PM proteins identified in control and DCD samples. NS, not significant; ∗*p* < 0.05; ∗∗*p* < 0.01; ∗∗∗*p* < 0.001 by unpaired two-tailed *t* test with Welch’s correction. iBAQ, intensity-based absolute quantification; PM, plasma membrane.

**Fig. 4:**
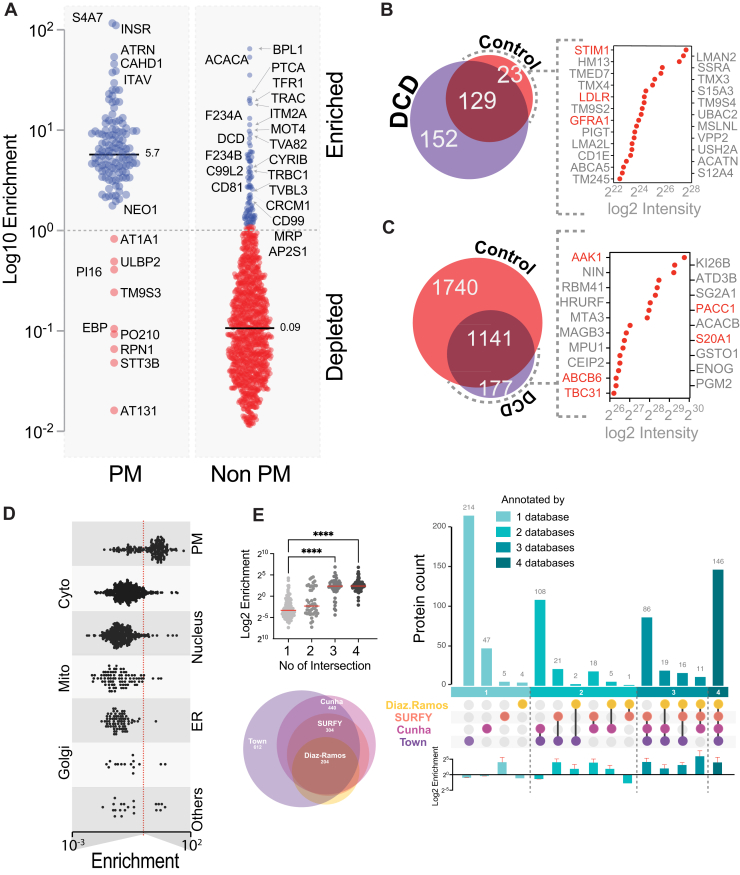
**Quantitative Enrichment Analysis Demonstrates Improved Specificity for Plasma Membrane Proteins Following Annexin V Dead Cell Depletion**. *A*, scatter plot displays enrichment scores calculated as the ratio of raw intensities in DCD *versus* control samples, with proteins annotated according to SURFY, where *blue circles* represent proteins with enrichment scores greater than 1 and *red circles* represent scores less than 1. *B* and *C*, the overlap of SURFY-annotated PM (*B*) and non-PM (*C*) proteins between control and DCD samples is shown. Raw intensities of the top PM proteins uniquely detected in control samples (*B*), or non-PM proteins uniquely detected in DCD samples (*C*) are shown on the *right*. Proteins in *red* indicate SURFY PM annotation, while those in *gray* are non-PM annotated. *D*, enrichment scores for proteins categorized by subcellular localization are compared based on UniProt annotations. *E*, multiple plots integrate PM annotation confidence across databases: the *top left* histogram depicts the number of proteins annotated in one or more databases alongside their enrichment scores; the *bottom left* Venn diagram shows overlaps of proteins identified in both control and DCD datasets and their presence in different PM annotation resources; The UpSet plot on the *right* depicts the overlap of proteins annotated by four external databases. *Vertical bars* indicate the number of proteins annotated by each combination of databases, from single-database annotations on the *left* to proteins annotated by all four databases on the *right*. The corresponding enrichment scores for each protein set are shown at the *bottom*. This visualization highlights both annotation overlap and functional enrichment simultaneously.

### Cell Lines and Primary Human Samples

Jurkat, HeLa, and HEK293 cells were obtained from the American Type Culture Collection. Primary human peripheral blood mononuclear cells (PBMCs) were isolated from leukopaks (StemCell Technologies) using immunomagnetic negative selection kits (BioLegend). Jurkat and primary PBMCs were cultured in RPMI-1640 medium supplemented with 10% fetal bovine serum (FBS), 1% penicillin-streptomycin-glutamine, and 0.1% 2-mercaptoethanol. HeLa and HEK293 cells were maintained in Dulbecco’s Modified Eagle Medium (DMEM) with 10% FBS and 1% penicillin-streptomycin-glutamine. All cultures were incubated at 37 °C in a humidified atmosphere containing 5% CO_2_.

### T Cell Isolation and Culture

PBMCs were isolated using the EasySep Direct Human PBMC Isolation Kit (StemCell Technologies), followed by negative selection of naïve CD4^+^ T cells using the MojoSort Human CD4 Naïve T Cell Isolation Kit (BioLegend). Isolated cells were cryopreserved in RPMI supplemented with 10% dimethyl sulfoxide and 10% FBS. For expansion, thawed CD4^+^ T cells were cultured in complete RPMI supplemented with 5 ng/ml recombinant human IL-7 and IL-15 (Thermo Fisher Scientific) for 14 days prior to biotinylation and flow cytometric analysis.

### Plasma Membrane Biotinylation

Surface protein labeling was performed using sulfo-NHS-based biotin reagents (Thermo Fisher Scientific) including sulfo-NHS-biotin, sulfo-NHS-LC-biotin, sulfo-NHS-SS-biotin, and sulfo-NHS-LCLC-biotin. Cells were washed twice with PBS supplemented with 0.5% FBS (PBS^sup^), then resuspended in PBS^sup^ at 1 × 10^8^ cells/ml. Biotin reagents (0.5 mM final concentration) were added and incubated at 4 °C for 20 min. Reactions were quenched by adding 50 mM Tris-HCl (pH 8.0) and incubated for 5 min at 4 °C. Cells were then washed twice in PBS and processed for flow cytometry or snap-frozen and stored at −80 °C.

### Annexin V Negative Selection (Dead Cell Depletion)

Following biotinylation, dead cells were removed using one of the following methods: fluorescence-activated cell sorting (FACS), Akadeum microbubble-based depletion, MojoSort Dead Cell Removal Kit (BioLegend), or the Dead Cell Removal Kit (Miltenyi Biotec) (13). FACS was performed by gating live cells on FSC-H vs. SSC-H. Manufacturer protocols were followed for all other methods. Postdepletion, viable cell counts and yields were recorded. The Miltenyi Dead Cell Removal Kit was used exclusively for all subsequent experiments after those shown in Figure 1*B*.

### Flow Cytometry and Imaging Cytometry

Flow cytometric analysis was performed using the NovoCyte Quanteon flow cytometer. Imaging flow cytometry was carried out on the ImageStreamX Mark II system. Surface and intracellular staining was performed in triplicate using antibodies against CD11a (clone HI111) and CD18 (clone CBR LFA-1/2) from BioLegend, and Calnexin (polyclonal, Abcam). Streptavidin-DyLight 649 and Streptavidin-FITC were used for detecting biotinylated proteins (Vector Laboratories). Cell viability was assessed using Annexin V and 7-AAD (BioLegend).

### Adherent-Cell-Surface Biotinylation and Confocal Imaging

HeLa cells were seeded onto precleaned glass coverslips and cultured until they reached approximately 80% confluence. Cells were washed twice with PBS and incubated with 500 μM Sulfo-NHS-Biotin (Thermo Fisher Scientific) for 20 min at 4 °C to label surface-accessible primary amines. Biotinylation was quenched by incubating cells with 50 mM Tris-HCl (pH 7.5) for 5 min at 4 °C, followed by two additional washes with ice-cold PBS. For staining, biotinylated cells were incubated with Streptavidin-DyLight-649 (10 μg/ml) together with DAPI (1 μg/ml) for 20 min at room temperature in the dark. After staining, coverslips were washed twice with PBS and once with ddH_2_O, then mounted onto glass slides using Mowiol 4 to 88 mounting medium and allowed to cure overnight at room temperature. Confocal images were acquired using a Zeiss LSM 900 microscope equipped with Airyscan 2 detection and a 63 × /1.4 NA oil-immersion objectives. DyLight-649 was excited with the 640-nm laser line and DAPI with the 405-nm laser. Emission was collected using optimized Airyscan detector settings and pixel dwell times recommended by the manufacturer. Images were acquired at Nyquist sampling, and the files were processed using ZEN Blue (v3.5.093, https://www.zeiss.com) using default settings. All imaging was performed under identical acquisition parameters between experimental conditions. Images were analyzed using Fiji (ImageJ, https://imagej.net/software/fiji). For each field, DAPI and Streptavidin-stained images were opened individually. DAPI images were converted to 8 bit grayscale, thresholded using the Yen method, and converted to binary masks to identify nuclei. Streptavidin images were smoothed with a Gaussian blur (σ = 1), thresholded using the Minimum method, and converted to binary masks to detect large, bright spots. Nuclei and Streptavidin-positive spots were counted manually using the “Analyze Particles” function with a size filter of 3000–Infinity pixels to exclude small background objects. The percentage of Biotin^high^ cells per field was calculated by normalizing the number of Streptavidin-positive spots to the total number of nuclei.

### Plasma Membrane Proteomics Sample Preparation

Frozen biotinylated cell pellets were lysed in 8 M urea buffer containing 0.3 M NaCl, 50 mM sodium phosphate (pH 8.0), 0.5% IGEPAL CA-630, and 1% SDS. Lysates (1 ml per 20 million cells) were homogenized via needle shearing (19G, 21G, and 23G), followed by 30 s sonication. Lysates were centrifuged (15,000×*g*, 15 min), and supernatants were incubated overnight at 4 °C with streptavidin agarose resin (Thermo Fisher Scientific; 112.5 μl slurry per mL of lysate).

Beads were washed five times with lysis buffer, followed by five washes in 8 M urea wash buffer (1.22 M NaCl, 0.5% SDS, 125 mM Tris-HCl pH 8.0, 10% ethanol, 10% isopropanol). Beads were washed with 25 mM ammonium bicarbonate (ABC), resuspended in 8 M urea in 25 mM ABC, and reduced with 5 mM TCEP (37 °C, 15 min), followed by alkylation with 10 mM iodoacetamide (RT, dark, 30 min). Beads were digested on-bead first with LysC (1.6 μg per 100 μl bead volume, 4 h at 37 °C), then diluted to 1.3 M urea and digested with trypsin (3.2 μg per 100 μl, 14 h at 37 °C). Supernatants were acidified (TFA to pH 3), desalted using C18 cartridges (preconditioned with acetonitrile (ACN), 50% ACN, 0.5% acetic acid, and 0.1% TFA), and eluted with 80% ACN. Eluates were dried via vacuum centrifugation and stored at −80 °C.

### Reversed-Phase Liquid Chromatography−Mass Spectrometry

Peptide mixtures were analyzed by LC-MS/MS using a Thermo Fisher Scientific UltiMate 3000 UHPLC system coupled online to a Thermo Fisher Scientific Orbitrap Fusion Lumos Tribrid Mass Spectrometer via a nano-electrospray ion source. Peptides were separated by reverse-phase chromatography on a 50 cm × 75 μm I.D. EasySpray Acclaim PepMap RSLC C18 analytical column (Thermo Fisher Scientific) using a linear 87-min gradient from 4% to 22% acetonitrile in 0.1% formic acid (solvent A: 0.1% formic acid in water; solvent B: 0.1% formic acid in acetonitrile) at a flow rate of 300 nl/min. Full MS scans were acquired in the Orbitrap over a mass range of 375 1500 m/z at a resolution of 60,000 (at m/z 400), followed by data-dependent MS/MS scans of the top 15 most intense precursor ions using higher collision-induced dissociation (HCD with a normalized collision energy of 30% in the linear ion trap. Dynamic exclusion was enabled with duration of 30 s. The AGC target was set to 4 × 10^5^ for MS1 and 6 × 10^e^ for MS2, with maximum injection times of 50 ms and 35 ms, respectively. Each sample was analyzed in technical duplicates to ensure reproducibility.

### Data Processing

Protein quantification from LC-MS/MS data was performed using MaxQuant software (version 2.1.3.0, https://www.maxquant.org) (12). Raw mass spectrometry files were searched against the human SwissProt database (22,355 protein entries; July 2024) (14). MS/MS spectra were filtered to retain a maximum of eight fragment peaks per 100 Da intervals. The precursor mass tolerance was set to 20 ppm for the initial search and 4.5 ppm for the main search, while fragment ions mass tolerance was set to 20 ppm. FDR for both peptide spectral matches and protein identifications were controlled at 1%, using the razor peptide approach. Trypsin was specified as the digestion enzyme, allowing up to two missed cleavages, with no allowance for nonspecific cleavage. Carbamidomethylation of cysteine residues was defined as a fixed modification, while methionine oxidation and N-terminal acetylation were set as variable modifications, allowing up to two variable modifications per peptide. Protein intensities were calculated as the integrated peak area across the chromatographic elution profile. For isotopic clusters, intensities from all isotopic peaks were summed. Only peptides classified as unique plus razor were used for quantification to ensure protein-level accuracy.

### Western Blotting

For SDS-PAGE, bead-bound proteins were eluted by boiling (95 °C, 20 min) in 1 × LDS sample buffer (Thermo Fisher Scientific). Supernatants were collected following centrifugation (1600 rpm, 5 min). Input and flow-through samples were quantified by UV absorbance and normalized to 20 μg per lane. Proteins were resolved on 4 to 12% Bis-Tris gels (Thermo Fisher Scientific) and transferred to membranes for immunoblotting. Antibodies were used against integrin β2 (D4N5Z), integrin αL (E5S9K), CD45 (D9M8I), CD7 (E4G1Q), Histone H3 (polyclonal), and β-actin (13E5) (Cell Signaling Technology), as well as PCCA (polyclonal, Abcam). Imaging was performed on an iBright FL1500 system (Invitrogen).

### Streptavidin Bead Flow Cytometry

Beads were washed twice with PBS, passed through a 70 μm cell strainer, centrifuged (400 × *g*, 10 min), and plated in 96-well round-bottom plates. Staining was performed using the same protocol as for cell-based flow cytometry. Antibodies against β-actin (SP124) and histone H3 (EPR16987) were obtained from Abcam.

### On-Bead Endoglycosidase Treatment

To assess glycoprotein status, streptavidin-bound proteins were treated with Endo H or PNGase F (New England Biolabs) following three PBS washes. Reactions were performed according to the manufacturer’s protocols. Beads were then washed with lysis buffer and processed for western blotting

## Results

### Amine-Labelling Produces Dead Cells with High Intracellular Protein Labels

For incompletely understood reasons, cell surface labelling and enrichment using lysine (amine) reactive biotinylation reagents results in contamination with intracellular proteins (15, 16, 17, 18, 19). Using flow cytometry to optimize cell surface biotinylation conditions of Jurkat T cells, we observed a bimodal distribution of streptavidin staining intensity (Fig. 1*A*). While ∼95% of cells were moderately labelled (biotin^mod^ cells), a small but distinct and reproducible population showed mean fluorescence intensity values that were ∼20 to 100-fold higher (biotin^high^ cells). Scatter profiles indicated that biotin^high^ cells had decreased forward scatter and increased side scatter, features consistent with nonviable Jurkat T cells. To directly examine cell viability, we performed 7-aminoactinomycin D (7-AAD) and Annexin V staining. 7-AAD is a membrane impermeable DNA-binding dye, which positively stains cells that have lost membrane integrity. Biotin^high^ cells were positive for markers, confirming compromised membrane integrity, and identifying them as dead or dying (Fig. 1*A*). To determine whether this labeling pattern is a general feature of amine-labelling reagents or unique to sulfo-NHS-biotin, we also tested the amine-reactive reagents sulfo-NHS-SS-biotin, sulfo-NHS-LC-biotin, and sulfo-NHS-LC-LC-biotin. All reagents tested resulted in the appearance of a biotin^high^ cell population at similar frequencies (Supplemental Fig. S1*A*). Fluorescence intensities of the biotin^high^ population remained approximately 20- to 100-fold higher than the biotin^mod^ population across all reagents (Fig. 1*B*).

Given the much higher abundance of intracellular proteins compared to PM proteins, the markedly higher labelling in these dead cells is consistent with intracellular biotinylation. To confirm and visualize intracellular labelling, we compared staining patterns in biotin^mod^ and biotin^high^ cells by imaging flow cytometry. Jurkat cells labeled with sulfo-NHS-biotin and stained with streptavidin-DyLight649, Annexin V-FITC, and the nuclear marker DAPI were imaged and analyzed. In biotin^mod^ cells, streptavidin signal localized predominantly to the PM. In contrast, biotin^high^ cells exhibited strong intracellular staining, with pronounced nuclear localization in many cells (Fig. 1*C* and Supplemental Fig. S1*B*). Co-localization analysis between streptavidin-DyLight649 and DAPI signals revealed a five-fold increase in similarity score in biotin^high^ cells, further supporting nuclear enrichment. Quantitative measurement using a DAPI-defined nuclear mask also showed a nearly five-fold increase in nuclear streptavidin signal in biotin^high^ *versus* biotin^mod^ cells (Fig. 1*D*).

We next assessed whether the intracellular biotinylation occurs in other cell types, including primary human T cells and adherent cells. Primary human CD4^+^ T cells isolated from leukopaks exhibited an even higher frequency of biotin^high^ cells (∼9%), likely due to reduced viability from sample transport and handling (Fig. 1*E*). Similarly, adherent HEK293 and HeLa cells labeled with sulfo-NHS-biotin also showed biotin^high^ populations by flow cytometry, though the frequency of this population varied (Fig. 1*E*). To confirm that intracellular labelling was not an artifact of detachment, we also performed confocal microscopy, which demonstrated exceptionally bright and intracellular staining in a minority of cells, consistent with the flow cytometry findings (Supplemental Fig. S2). These findings suggest that intracellular labeling of nonviable cells is a general phenomenon of amine-reactive biotinylation protocols across diverse cell types. Given the ∼20-100-fold higher labelling observed in these events, our data also suggest that a small population of nonviable cells can contribute disproportionately to contamination, consistent with previous reports highlighting significant intracellular and nuclear contamination in amine-labelling surface proteomics workflows (20, 21).

### Biotin^high^ Cells can be Efficiently Depleted Post-biotinylation

We next examined whether altering labelling conditions or cell culture conditions could reduce the biotin^high^ population. Titrating reagent concentrations revealed that the biotin^high^ population was detectable even when biotinylating at 0.0625 mM for all biotin reagents tested (Supplemental Fig. S3*A*). Similarly, reducing the biotinylation reaction time resulted in a modest decrease in fluorescence intensity but did not significantly alter the percentage of biotin^high^ cells (Suppletmenal Fig. S3*B*). Finally, we examined the impact of improving cell viability by reducing cell culture density, on the frequency of biotin^high^ cells. The American Type Culture Collection recommends growing Jurkat cells up to 3 x 10^6^ cells per ml. We routinely culture Jurkat cells up to 2 x 10^6^ cells per ml. As expected, increasing cell density further led to more cell death and increased proportions of biotin^high^ cells. Reducing cell culturing density moderately reduced the proportion of biotin^high^ cells, but failed to eliminate them entirely (Supplemental Fig. S3*B*).

We thus turned our attention to removal of these cells postlabelling. Since biotin^high^ cells are Annexin V positive (Fig. 1*A*), we hypothesized that Annexin V based negative selection could effectively deplete dead cells and reduce intracellular labeling. Commercially, several Annexin V based methods are available for dead cell removal. In addition, as shown previously (Fig. 1*A*), biotin^high^ cells exhibit distinct FSC/SSC profiles, enabling separation by FACS (22). FACS sorting based on FSC/SSC alone efficiently reduced biotin^high^ cells from 4.5% to 0.23%. Although additional sorting based on viability markers would likely improve this further, FACS sorting was slow, required specialized equipment, and yielded low recovery (∼25%) (Fig. 2*B*).

We therefore turned our attention to three commercially available Annexin V–based kits: Miltenyi magnetic-activated cell sorting (MACS) beads, Akadeum microbubbles, and the BioLegend Dead Cell Removal Kit. These approaches have the advantage of processing large numbers of cells quickly, increasing potential suitability for downstream proteomics. Among them, MACS achieved the highest depletion efficiency, reducing biotin^high^ cells from 4.5% to 0.03%, with ∼60% recovery. The BioLegend kit reducing dead cells to 0.95%, while the microbubble method showed better depletion but also more cell loss (∼40% recovery) (Fig. 2*B*). Given the highly effective depletion of biotin^high^ cells, short processing times, and relatively high cell recovery, we proceeded with the MACS approach as our standard dead cell depletion method.

We then compared pre*versus* postbiotinylation dead cell depletion. While depleting dead cells prior to sulfo-NHS-biotin labeling modestly reduced the biotin^high^ population, it was notably less effective than dead cell depletion postlabelling (Fig. 2*C*). This suggests that the labeling itself or the general processing of the cells continually induces cell death. Indeed, a similar number of 7-AAD^+^Annexin V^+^ cells persisted in both conditions. We also examined the effectiveness of dead cell depletion in primary human T cells. Growing primary human T cells in the presence of the pro-survival cytokines IL-7 and IL-15 for 2 weeks reduced the number of biotin^high^ cells to ∼2%. However, like the Jurkat cells, dead cell depletion using the MACS method, reduced this number further to 0.06%, essentially eliminating biotin^high^ events (Supplemental Fig. S3*C*). Overall, these data demonstrate that sulfo-NHS-biotin labeling bypasses the PM in a subset of cells and that postlabeling dead cell depletion can efficiently remove these intracellularly labelled biotin^high^ events.

### Dead Cell Depletion Reduces Intracellular Contaminants and Improves Surfaceomics

We expected the removal of intracellularly biotinylated cells to deplete intracellular contaminants and improve downstream enrichment of cell surface proteins. To first estimate the extent of this improvement on surface proteome quality, we compared streptavidin bead capture (IP) for key intracellular and PM proteins from NHS-biotin-labeled samples, with or without dead cell depletion. Postlabeling dead cell depletion dramatically reduced the detection of histone H3 and β-actin by western blotting of captured proteins, while enhancing the enrichment of the known Jurkat plasma membrane proteins CD7, CD45 and integrin β2 (Fig. 2*D* and Supplemental Fig. S3*D*).

Next, we evaluated the effect of dead cell depletion on cell surface proteomic profiling by mass spectrometry (Supplemental Table S1). Dead cell depletion resulted in fewer total proteins (1599 *versus* 3033) and peptides (12,687 *versus* 24,157) than the control sample (Fig. 3, *A*–*B*, Supplemental Table S2). Despite this however, it markedly enhanced enrichment of PM proteins. Using SURFY (https://github.com/Bribak/SURFY2) annotations (Supplemental Table S3) (7), 281 PM proteins were identified with dead cell depletion, compared to 152 in the control sample, representing an 85% increase. As a proportion of total ID’s, bona fide PM ID’s increased from 5.1% to 17.6% with dead cell depletion (Fig. 3*C*). In addition, the PM proteins identified had dramatically higher peptide coverage and were present at much higher intensities (Fig. 3, *D*–*E*). Concurrently, the peptide coverage and intensities of nonPM proteins were markedly reduced with dead cell depletion consistent with reduced intracellular contamination (Fig. 3, *D*–*E*). A second independent surfaceomics replicate was performed and showed consistent plasma membrane protein enrichment (Supplemental Fig. S4, *A*–*C*).

As an aggregate measure of PM enrichment, we examined the cumulative proportional intensities of all PM and nonPM proteins. The aggregate proportional intensity of all PM proteins increased from 4% in the control sample to 55.8% with dead cell depletion, indicating a ∼14-fold enrichment in PM proteins (Fig. 3*F*). By contrast, total intracellular protein intensity decreased from 96% to 44.2% (Fig. 3*G*).

We next examined the top 10 most abundant proteins in each condition. Without dead cell depletion, the top-ranking proteins were predominantly high-abundance intracellular contaminants such as nuclear histones and cytoplasmic actin (Fig. 3*H*). In contrast, the most abundant proteins with dead cell depletion were PM-localized, with the exception of two endogenously biotinylated mitochondrial proteins (ACACA and PYC) (Fig. 3*I*) (23, 24). These findings suggest that without dead cell depletion, proteomic profiles are biased toward abundant intracellular contaminants, obscuring true surface protein enrichment.

To visualize broader trends in proteome composition, we mapped all quantified proteins by summed intensity and annotated structural features. In dead cell depleted samples, PM proteins formed a distinct and concentrated cluster, in stark contrast to the scattered distribution observed in control samples (Fig. 3, *J*–*K*). Additionally, dead cell depletion produced a greater enrichment of Type I transmembrane proteins (purple rectangles) and GPI-anchored proteins (green diamonds) (Fig. 3, *J*–*K*, Supplemental Table S3). Multi-pass transmembrane proteins (blue triangles) were comparably represented in both conditions. These results indicate that dead cell depletion preferentially enriches proteins with canonical cell surface features (14). The lower enrichment of multipass membrane proteins in our dataset may be attributable to the high hydrophobicity of their transmembrane peptides, which can reduce solubilization efficiency and peptide recovery during sample preparation and LC-MS/MS analysis (25, 26).

Gene Ontology enrichment analysis further supported these findings. The control data set was significantly enriched for nuclear, cytoplasmic, and mitochondrial proteins, indicative of pervasive intracellular contamination (Fig. 3*L*) (14, 27). Conversely, dead cell depletion demonstrated strong enrichment for proteins localized to the plasma membrane, extracellular space, and endosomal compartments, with high statistical significance (FDR <0.05) (Fig. 3*M*). These shifts confirm the enhanced specificity of PM proteomes derived by dead cell depletion (14, 27).

To investigate whether transcript abundance influences protein detection in our datasets, we analyzed gene expression profiles of Jurkat E6-1 cells using publicly available RNA-seq data from the Human Protein Atlas (Supplemental Table S2) (28). PM proteins uniquely identified in control samples exhibited significantly higher transcript levels compared to those exclusively detected in Annexin V-based dead cell–depleted samples. This suggests that in control samples, highly expressed PM proteins dominate the profile, potentially due to signal dilution from substantial contamination by abundant intracellular proteins. In contrast, the reduction of intracellular proteins by dead cell depletion facilitated the detection of lower-abundance PM proteins (Fig. 3*N*). Among the identified PM proteins, those found only with dead cell depletion had significantly lower transcript levels compared to PM proteins detected in control samples or in both conditions (Supplemental Fig. S4*D*). Collectively, these data demonstrate that Annexin V–based dead cell depletion significantly improves both the specificity and sensitivity of plasma membrane proteomic profiling by reducing intracellular contamination and enhancing the detection of lower-abundance surface proteins.

### Quantitative Scoring Reveals Specific Enrichment of Surface-Localized Proteins with Dead Cell Depletion

To better characterize the specificity of PM protein enrichment with dead cell depletion, we calculated a quantitative enrichment score based on the ratio of protein intensities between dead cell depleted and control samples. This metric allowed us to assess the degree of enrichment for each protein across conditions. Among 129 SURFY annotated PM proteins identified in both samples, the mean enrichment score was 5.7. Notably, 120 of these proteins had an enrichment score greater than 1, indicating increased abundance with dead cell depletion. The remaining nine proteins with scores <1 were either intracellular proteins or proteins with ambiguous localization. For instance, EBP, PO210, RPN1, STT3B, and ATP13A1 are predominantly intracellular, while ATP1A1, ULBP2, and TM9SF3 are annotated as both PM and intracellular in various databases (Fig. 4*A*, Supplemental Table S2) (29, 30, 31, 32). In contrast, proteins annotated as nonPM had a mean enrichment score of 0.06. Of these, 1045 had scores <1, and only 96 exceeded 1 further supporting selective depletion of intracellular proteins. Interestingly, two proteins with the highest enrichment scores, BPL1 (score = 66.6) and ACACA (score = 54.6) are known to be endogenously biotinylated (BPL1 catalyzes the biotinylation of carboxylases and histones, while ACACA is a biotin-containing enzyme), likely accounting for their elevated scores (33, 34). Excluding such exceptions, approximately 20 high-scoring proteins not annotated by SURFY were found to be PM proteins in other databases (14, 35), including well established PM proteins such as transferrin receptor (TFR1) and components of the T cell receptor complex (TRAC, TRBC1 and TVBL3) (Fig. 4*A*). These findings suggest that despite annotation ambiguities, dead cell depletion effectively enriches bona fide surface proteins.

We next examined proteins that were only identified in the control or dead cell depletion samples. The 152 unique proteins with SURFY PM annotation in the dead cell depleted sample were also consistently annotated as PM proteins in other databases (14, 35). By contrast, of the 23 unique proteins with SURFY PM annotation in the control sample (Fig. 4*B*, Supplemental Table S4), the majority were consistent with nonPM localization (7, 14). Conversely, 177 unique proteins annotated as nonPM by SURFY were identified in the dead cell depleted sample, of which ∼30 were nevertheless localized to or associated with the PM (7, 14). These findings further reinforce the specificity of the dead cell depletion strategy (Fig. 4*C*, Supplemental Table S4).

Given the variability in PM annotations across databases, we next evaluated the subcellular localization of proteins using UniProt (14). We plotted enrichment scores by UniProt annotations and found that PM-localized proteins had the highest mean enrichment score (6.7), whereas proteins localized to the cytosol, nucleus, mitochondria, ER, or Golgi had mean scores <1 (Fig. 4*D*, Supplemental Table S5). This result is consistent with selective PM enrichment in dead cell depleted samples and reduced contamination from intracellular compartments. To assess whether enrichment scores correlated with annotation confidence, we integrated four PM databases (Diaz-Ramos, Bausch-Fluck, Town, and Cunha) and categorized proteins based on the number of databases supporting their PM localization (7, 36, 37, 38). We identified 454 PM proteins present in our dataset that were annotated in at least one of the databases. Proteins annotated in only one database (n = 113) had a mean enrichment score of 1.6. Those annotated in two databases (n = 79) had a mean score of 5.6; three databases (n = 119) yielded a score of 8.4; and proteins supported by all four databases (n = 79) had a mean enrichment score of 8.3 (Fig. 4*E*, Supplemental Table S5). We analyzed the annotation of all quantified proteins across four databases to assess enrichment patterns. Among proteins annotated by a single database, SURFY showed the highest enrichment (n = 5). For proteins annotated by two databases, the combination SURFY-Town (n = 21) and Cunha-SURFY (n = 18) exhibited the greatest enrichment. In the set of proteins annotated by three databases, Diaz.Ramos–SURFY-Cunha (n = 11) displayed the highest enrichment score (Fig. 4*E*). Consistent with previous observations, proteins annotated by a greater number of databases generally exhibited higher enrichment. This trend suggests a positive correlation between enrichment score and the confidence level of PM localization. Taken together, these results indicate that dead cell depletion not only improves sensitivity for detecting PM proteins, but also selectively enriches high-confidence surface-localized proteins.

### Dead Cell Depletion Specifically Removes the Intracellular Pool of PM Proteins

**Fig. 5:**
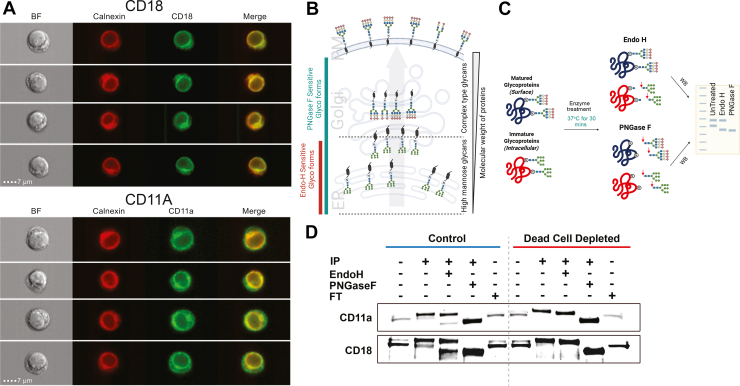
**Dead Cell Depletion Selectively Removes Intracellular Pools of Plasma Membrane Proteins to Enhance Surfaceome Specificity**. *A*, representative images of Jurkat cells showing intracellular localization of CD18 (ITGB2) and CD11 A (ITGAL) in relation to the ER marker calnexin. Cells were fixed, permeabilized, and stained with CD18-FITC or CD11A-FITC, and calnexin, then analyzed using the ImageStreamX Mark II imaging flow cytometer to visualize subcellular distribution. *B*, schematic illustration of N-glycan maturation along the secretory pathway, is highlighting the presence of Endo H–sensitive (ER-resident) and Endo H–resistant (post-Golgi) glycoforms. *C*, overview of the experimental strategy used to distinguish intracellular *versus* plasma membrane pools of biotin-enriched proteins. *D*, Dead cell depletion reduces intracellular contamination in the biotin-enriched fraction. Biotin-labeled proteins from control and DCD samples were enriched using streptavidin agarose beads and treated with either Endo H or PNGase F overnight at 37 °C. Posttreatment, proteins were eluted and analyzed by SDS-PAGE followed by Western blotting using primary antibodies against CD18 and CD11 A, and HRP-conjugated secondary antibodies.

PM proteins reside in both intracellular and cell-surface pools, and distribution between these compartments can be dynamically regulated (39). For example, glucose transporters are stored intracellularly in the absence of insulin, but are quickly trafficked to the cell surface in response to insulin signaling (40). Based on our findings that sulfo-NHS-biotin labels intracellular proteins in biotin^high^ dead cells, we hypothesized that routine labelling may capture both cell-surface and intracellular pools of PM proteins and that dead cell depletion will selectively remove the intracellular contribution to more faithfully reflect the cell surface proteome. To test this hypothesis, we examined LFA-1. LFA-1 is a heterodimer composed of integrin αL (CD11a) and integrin β2 (CD18) (41). It is a critical cell adhesion molecule expressed on leukocytes which has also been shown to have an intracellular pool in T cells (42). We first tested whether cell surface and intracellular pools could be detected in Jurkat T cells by imaging flow cytometry. Indeed, both CD11a and CD18 demonstrated extensive intracellular staining and showed at least partial co-localization with the ER marker Calnexin (Fig. 5, *A*–*B*).

To distinguish cell surface and intracellular pools, we leveraged selective deglycosylation by Endo H, an endoglycosidase which specifically cleaves immature N-glycans (high mannose and hybrid N-glycans) but not Golgi remodeled N-glycans (complex N-glycans) (Fig. 5*C*). As a control, we also used PNGase F, which cleaves essentially all N-glycans. Both CD11a and CD18 demonstrated two distinct bands by western blotting (Fig. 5*D*) (43). In control whole cell lysate (input), the lower molecular weight (MW) species was more abundant. However, following cell surface enrichment, the higher MW band became more abundant. This is consistent with the lower molecular weight species representing the large intracellular ER pool while the higher molecular weight band representing the cell surface glycoform that is enriched with cell surface biotinylation. Indeed, while PNGase F collapsed both bands to a lower molecular weight deglycosylated form (Fig. 5*D*, lane 4), Endo H only deglycosylated the lower molecular weight species (Fig. 5*D*, lane 3), confirming that the lower MW band represents an immature glycoform. Critically, comparison of the streptavidin enriched IP fractions demonstrated that dead cell depletion results in selective loss of the Endo H sensitive, lower molecular weight bands of both CD11a and CD18 (Fig. 5*D*, lanes 7–9) (44, 45). These data provide proof of concept that intracellular labelling in biotin^high^ cells leads to contamination of the subsequently enriched surfaceome with intracellular sources of PM proteins, and that dead cell depletion can remove these contaminants to more accurately reflect the surfaceome.

## Discussion

Amine-reactive chemical labeling approaches, such as sulfo-NHS-biotin, are widely used in cell surface proteomics due to their ability to covalently modify accessible primary amines on extracellular lysines (9, 15, 16, 19). However, our data demonstrate that these reagents also biotinylate intracellular proteins in nonviable cells, introducing a major source of contamination. Despite comprising a small fraction of the total cell population, nonviable biotin^high^ cells exhibit 20- to 100-fold higher biotin signal than viable cells due to the substantially greater abundance of intracellular proteins (46) (Fig. 1). This disproportionate labeling can dominate downstream surface-enriched proteomes, obscuring the detection of bona fide plasma membrane (PM) proteins (Fig. 2, Fig. 3). Indeed, assuming a somewhat arbitrary but convenient middle point of 50-fold higher labeling in dead cells illustrates this point. In this case, even 2% dead cells would contribute as much protein as the 98% of surface labelled cells (2% x 50 = 100 arbitrary units; 98% x 1 = 98 arbitrary units).

While our optimization of cell culture conditions and prelabeling depletion of dead cells modestly reduced biotin^high^ contamination, removing Annexin V+ cells after sulfo-NHS-biotin labeling was highly effective. This dead cell depletion essentially eliminated the biotin^high^ population and significantly reduced intracellular protein contamination, as confirmed by decreased abundance of canonical intracellular markers such as histones and actin and improves the detection of transmembrane and GPI-anchored PM proteins (Fig. 2, Fig. 3). This strategy increases not only the number of uniquely identified PM proteins but also enhances their peptide coverage and signal intensity, thereby improving both the specificity and quality of surfaceome analyses (Fig. 3). We have not ruled out however, that intracellular biotinylation may be reduced or eliminated by other approaches. Improvements in cell viability or dead cell removal by other methods are predicted to improve downstream surfaceomics proportional to the extent of their biotin^high^ cell depletion.

Furthermore, the value of dead cell depletion may vary depending on the cell type, culture/labelling conditions, and possibly other experimental details. As shown, flow cytometry, imaging flow cytometry, and or confocal microscopy can be used to evaluate the extent of dead cell labelling in other experimental systems and workflows. As the percentage of biotin^high^ cells and their mean fluorescence intensity increases, so does the expected value of dead cell depletion. Our imaging and flow cytometry analyses demonstrate that intracellular biotin labeling is not restricted to Jurkat cells. We also observed intracellularly labelled biotin^high^ cells in primary human T cells, HeLa, and HEK293 cells, supporting the generalizability of these findings (Supplemental Fig. S2). However, our proteomic analysis was performed only in Jurkat cells. Therefore, the extent to which our surfaceome improvements translate to other cell types remains to be determined, particularly for adherent cells where washing may reduce dead cells.

Our data also show that intracellular labeling of dead cells includes not just cytoplasmic and nuclear proteins, but also intracellular pools of PM proteins (Fig. 5). This underscores a key limitation of surfaceome studies that rely solely on compartment-based annotation for filtering (47, 48). While annotation-based filtering can remove strictly intracellular proteins, it cannot distinguish between intracellular and surface-resident pools of proteins with dual localization, such as trafficking PM proteins (GLUT4) or proteins enroute through the secretory pathway (TFR1) (40, 49).

Our quantitative enrichment scoring further supports the ability of Annexin V based depletion to enrich for high-confidence PM proteins, particularly those supported by multiple surfaceome databases (7, 36, 37, 38). Conversely, many proteins detected exclusively in control samples appear to be misannotated or derive from abundant intracellular compartments, highlighting the potential for dead cell depletion to refine surface protein annotations and reduce false positives in surfaceome datasets (Fig. 4) (7, 36, 37, 38).

Although our study specifically investigates amine-reactive labeling using sulfo-NHS-biotin, the findings may apply to other chemistries. Many labeling chemistries, whether targeting lysines, thiols, or glycans rely on intact membrane integrity to ensure surface specificity (50). Intracellular contamination is, therefore, a general concern in chemical surface labeling and should be examined. The extent of contamination may depend on the distribution of reactive functional groups, which are typically more abundant intracellularly due to higher total protein content. For instance, lysine residues are proportionally more prevalent in intracellular proteins, which likely explains the strong intracellular signal in biotin^high^ cells (51). In contrast, glycan-based labeling strategies may show less intracellular labeling, as complex glycans are largely absent from the cytoplasm and nucleus (52). However, even glycan-reactive labels are not immune to contamination from intracellular pools of PM proteins. Indeed, glycan-based datasets frequently include ER- and Golgi-resident glycoproteins such as oligosaccharyltransferase complex members and glycosyltransferases, suggesting that nonviable cells may contribute artifacts even in these workflows (53).

Our findings highlight an important consideration for quantitative surfaceomics studies aiming to compare cell surface expression across conditions. Without dead cell depletion, dynamic changes in surface abundance may be masked by variable levels of intracellular contamination, especially for proteins with both surface and intracellular pools. This can result in misleading interpretations of differential expression or trafficking dynamics. Dead cell depletion is thus expected to enhance the quantitative accuracy of surface expression changes by decreasing the contribution of intracellular pools of PM proteins.

In summary, we identify postlabeling dead cell depletion as a critical step for improving the quality of amine-based surface proteomics. By effectively removing biotin^high^ nonviable cells, Annexin V-based negative selection enhances the detection of true PM proteins, reduces intracellular contamination, and increases the reliability of both qualitative and quantitative analyses. As surfaceome studies continue to expand in scale and application from immunophenotyping to biomarker and therapeutic target discovery, standardizing approaches to mitigate dead cell-driven artifacts will be essential (54, 55). Furthermore, extending these findings to other labeling chemistries will be an important avenue for future investigation aimed at further improving cell-surface-specific proteomic profiling.

## Data Availability

The mass spectrometry proteomics data have been deposited to the ProteomeXchange Consortium (http://proteomecentral.proteomexchange.org) via the PRIDE partner repository with the dataset identifier PXD068422"

## Supplemental Data

This article contains supplemental data.

## Declaration of Generative AI and AI-Assisted Technologies

No AI or AI-assisted technologies were used in this work.

## Conflictof Interests

The authors declare no competing interests.

## References

[1] Alberts B. (2002). Molecular Biology of the Cell.

[2] Overington J.P., Al-Lazikani B., Hopkins A.L. (2006). How many drug targets are there?. Nat. Rev. Drug Discov..

[3] Santos R., Ursu O., Gaulton A., Bento A.P., Donadi R.S., Bologa C.G. (2017). A comprehensive map of molecular drug targets. Nat. Rev. Drug Discov..

[4] Helenius A., Aebi M. (2004). Roles of N-Linked glycans in the endoplasmic reticulum. Annu. Rev. Biochem..

[5] Lodish H., Berk A., Kaiser C., Krieger M. (2016). Molecular Cell Biology.

[6] Maxfield F.R., McGraw T.E. (2004). Endocytic recycling. Nat. Rev. Mol. Cell. Biol..

[7] Bausch-Fluck D., Goldmann U., Müller S., Van Oostrum M., Müller M., Schubert O.T. (2018). The in silico human surfaceome. Proc. Natl. Acad. Sci. U. S. A..

[8] Wu C.C., Yates J.R. (2003). The application of mass spectrometry to membrane proteomics. Nat. Biotechnol..

[9] Hörmann K., Stukalov A., Müller A.C., Heinz L.X., Superti-Furga G., Colinge J. (2016). A surface biotinylation strategy for reproducible plasma membrane protein purification and tracking of genetic and drug-induced alterations. J. Proteome Res..

[10] Weekes M.P., Antrobus R., Lill J.R., Duncan L.M., Hör S., Lehner P.J. (2010). Comparative analysis of techniques to purify plasma membrane proteins. J. Biomol. Tech..

[11] Karhemo P.-R., Ravela S., Laakso M., Ritamo I., Tatti O., Mäkinen S. (2012). An optimized isolation of biotinylated cell surface proteins reveals novel players in cancer metastasis. J. Proteomics..

[12] Tyanova S., Temu T., Cox J. (2016). The MaxQuant computational platform for mass spectrometry-based shotgun proteomics. Nat. Protoc..

[13] Logue S.E., Elgendy M., Martin S.J. (2009). Expression, purification and use of recombinant annexin V for the detection of apoptotic cells. Nat. Protoc..

[14] Ahmad S., Jose da Costa Gonzales L., Bowler-Barnett E.H., Rice D.L., Kim M., Wijerathne S. (2025). The UniProt website API: facilitating programmatic access to protein knowledge. Nucleic Acids Res..

[15] Li Y., Qin H., Ye M. (2020). An overview on enrichment methods for cell surface proteome profiling. J. Separation Sci..

[16] Bausch-Fluck D., Hofmann A., Wollscheid B., Josic D., Hixson D.C. (2012). Liver Proteomics, Methods in Molecular Biology.

[17] Elschenbroich S., Kim Y., Medin J.A., Kislinger T. (2010). Isolation of cell surface proteins for mass spectrometry-based proteomics. Expert Rev. Proteomics.

[18] Griffin N.M., Schnitzer J.E. (2011). Overcoming key technological challenges in using mass spectrometry for mapping cell surfaces in tissues. Mol. Cell. Proteomics.

[19] Kuhlmann L., Cummins E., Samudio I., Kislinger T. (2018). Cell-surface proteomics for the identification of novel therapeutic targets in cancer. Expert Rev. Proteomics.

[20] Aggelis V., Craven R.A., Peng J., Harnden P., Cairns D.A., Maher E.R. (2009). Proteomic identification of differentially expressed plasma membrane proteins in renal cell carcinoma by stable isotope labelling of a von Hippel-Lindau transfectant cell line model. Proteomics.

[21] Qiu H., Wang Y. (2008). Quantitative analysis of surface plasma membrane proteins of primary and metastatic melanoma cells. J. Proteome Res..

[22] Ormerod M.G., Paul F., Cheetham M., Sun X.-M. (1995). Discrimination of apoptotic thymocytes by forward light scatter. Cytometry.

[23] Tong L. (2013). Structure and function of biotin-dependent carboxylases. Cell. Mol. Life Sci..

[24] Báez-Saldaña A., Zendejas-Ruiz I., Revilla-Monsalve C., Islas-Andrade S., Cárdenas A., Rojas-Ochoa A. (2004). Effects of biotin on pyruvate carboxylase, acetyl-CoA carboxylase, propionyl-CoA carboxylase, and markers for glucose and lipid homeostasis in type 2 diabetic patients and nondiabetic subjects. Am. J. Clin. Nutr..

[25] Eichacker L.A., Granvogl B., Mirus O., Müller B.C., Miess C., Schleiff E. (2004). Hiding behind hydrophobicity. J. Biol. Chem..

[26] Vit O., Petrak J. (2017). Integral membrane proteins in proteomics. How to break open the black box?. J. Proteomics..

[27] Watson J., Smith M., Francavilla C., Schwartz J.-M. (2022). SubcellulaRVis: a web-based tool to simplify and visualise subcellular compartment enrichment. Nucleic Acids Res..

[28] Uhlen M., Karlsson M.J., Zhong W., Tebani A., Pou C., Mikes J. (2019). A genome-wide transcriptomic analysis of protein-coding genes in human blood cells. Science.

[29] Sato T., Sako Y., Sho M., Momohara M., Suico M.A., Shuto T. (2012). STT3B-Dependent posttranslational N-Glycosylation as a surveillance System for secretory protein. Mol. Cell.

[30] McKenna M.J., Sim S.I., Ordureau A., Wei L., Harper J.W., Shao S. (2020). The endoplasmic reticulum P5A-ATPase is a transmembrane helix dislocase. Science.

[31] Lingemann M., McCarty T., Liu X., Buchholz U.J., Surman S., Martin S.E. (2019). The alpha-1 subunit of the Na+,K+-ATPase (ATP1A1) is required for macropinocytic entry of respiratory syncytial virus (RSV) in human respiratory epithelial cells. PLoS Pathog..

[32] Yang J., Dong Y., Xu J., Qian X., Cai Y., Chen Y. (2025). TM9SF3 is a Golgi-resident ATG8-binding protein essential for Golgi-selective autophagy. Dev. Cell.

[33] Watts J.S., Morton D.G., Kemphues K.J., Watts J.L. (2018). The biotin-ligating protein BPL-1 is critical for lipid biosynthesis and polarization of the Caenorhabditis elegans embryo. J. Biol. Chem..

[34] Keereetaweep J., Liu H., Zhai Z., Shanklin J. (2018). Biotin attachment domain-containing proteins irreversibly inhibit acetyl CoA carboxylase. Plant Physiol..

[35] Binder J.X., Pletscher-Frankild S., Tsafou K., Stolte C., O’Donoghue S.I., Schneider R. (2014). COMPARTMENTS: unification and visualization of protein subcellular localization evidence. Database.

[36] Díaz-Ramos M.C., Engel P., Bastos R. (2011). Towards a comprehensive human cell-surface immunome database. Immunol. Lett..

[37] Town J., Pais H., Harrison S., Stead L.F., Bataille C., Bunjobpol W. (2016). Exploring the surfaceome of ewing sarcoma identifies a new and unique therapeutic target. Proc. Natl. Acad. Sci. U. S. A..

[38] Da Cunha J.P.C., Galante P.A.F., De Souza J.E., De Souza R.F., Carvalho P.M., Ohara D.T. (2009). Bioinformatics construction of the human cell surfaceome. Proc. Natl. Acad. Sci. U. S. A..

[39] Van Oostrum M., Campbell B., Seng C., Müller M., Tom Dieck S., Hammer J. (2020). Surfaceome dynamics reveal proteostasis-independent reorganization of neuronal surface proteins during development and synaptic plasticity. Nat. Commun..

[40] Cheatham B., Volchuk A., Kahn C.R., Wang L., Rhodes C.J., Klip A. (1996). Insulin-stimulated translocation of GLUT4 glucose transporters requires SNARE-complex proteins. Proc. Natl. Acad. Sci. U. S. A..

[41] Nordenfelt P., Moore T.I., Mehta S.B., Kalappurakkal J.M., Swaminathan V., Koga N. (2017). Direction of actin flow dictates integrin LFA-1 orientation during leukocyte migration. Nat. Commun..

[42] Capece T., Walling B.L., Lim K., Kim K.-D., Bae S., Chung H.-L. (2017). A novel intracellular pool of LFA-1 is critical for asymmetric CD8+ T cell activation and differentiation. J. Cell Biol..

[43] Tarentino A.L., Plummer T.H. (1994). Methods in Enzymology.

[44] Wang G., de Jong R.N., van den Bremer E.T.J., Parren P.W.H.I., Heck A.J.R. (2017). Enhancing accuracy in molecular weight determination of highly heterogeneously glycosylated proteins by native Tandem Mass spectrometry. Anal Chem..

[45] Stanley P. (2011). Golgi glycosylation. Cold Spring Harbor Perspect. Biol..

[46] Garapati K., Ding H., Charlesworth M.C., Kim Y., Zenka R., Saraswat M. (2023). sBioSITe enables sensitive identification of the cell surface proteome through direct enrichment of biotinylated peptides. Clin. Proteomics.

[47] Sprenger J., Lynn Fink J., Karunaratne S., Hanson K., Hamilton N.A., Teasdale R.D. (2007). LOCATE: a mammalian protein subcellular localization database. Nucleic Acids Res..

[48] Aturaliya R.N., Fink J.L., Davis M.J., Teasdale M.S., Hanson K.A., Miranda K.C. (2006). Subcellular localization of Mammalian type II membrane proteins. Traffic.

[49] Mayle K.M., Le A.M., Kamei D.T. (2012). The intracellular trafficking pathway of transferrin. Biochim. Biophys. Acta. Gen. Subjects.

[50] Kirkemo L.L., Elledge S.K., Yang J., Byrnes J.R., Glasgow J.E., Blelloch R. (2022). Cell-surface tethered promiscuous biotinylators enable comparative small-scale surface proteomic analysis of human extracellular vesicles and cells. Elife.

[51] Kim S.C., Sprung R., Chen Y., Xu Y., Ball H., Pei J. (2006). Substrate and functional diversity of lysine acetylation Revealed by a Proteomics Survey. Mol. Cell.

[52] Wollscheid B., Bausch-Fluck D., Henderson C., O’Brien R., Bibel M., Schiess R. (2009). Mass-spectrometric identification and relative quantification of N-linked cell surface glycoproteins. Nat. Biotechnol..

[53] Kalxdorf M., Gade S., Eberl H.C., Bantscheff M. (2017). Monitoring cell-surface N-Glycoproteome dynamics by quantitative proteomics reveals mechanistic insights into macrophage differentiation. Mol. Cell. Proteomics.

[54] Pauwels J., Fijałkowska D., Eyckerman S., Gevaert K. (2022). Mass spectrometry and the cellular surfaceome. Mass Spectrom. Rev..

[55] Nowak-Terpiłowska A., Śledziński P., Zeyland J. (2021). Impact of cell harvesting methods on detection of cell surface proteins and apoptotic markers. Braz. J. Med. Biol. Res..

