# Supplementary material for: Improved T Cell Surfaceomics by Depleting Intracellularly Labelled Dead Cells
Source: Mol Cell Proteomics. 2025 Dec 24;25(2):101503. doi: 10.1016/j.mcpro.2025.101503 (PMC12859484; doi:10.1016/j.mcpro.2025.101503)
Supplement: Supplemental — Data [file mmc6.docx]

**Supplementary Figures**

**Fig. S1**

**
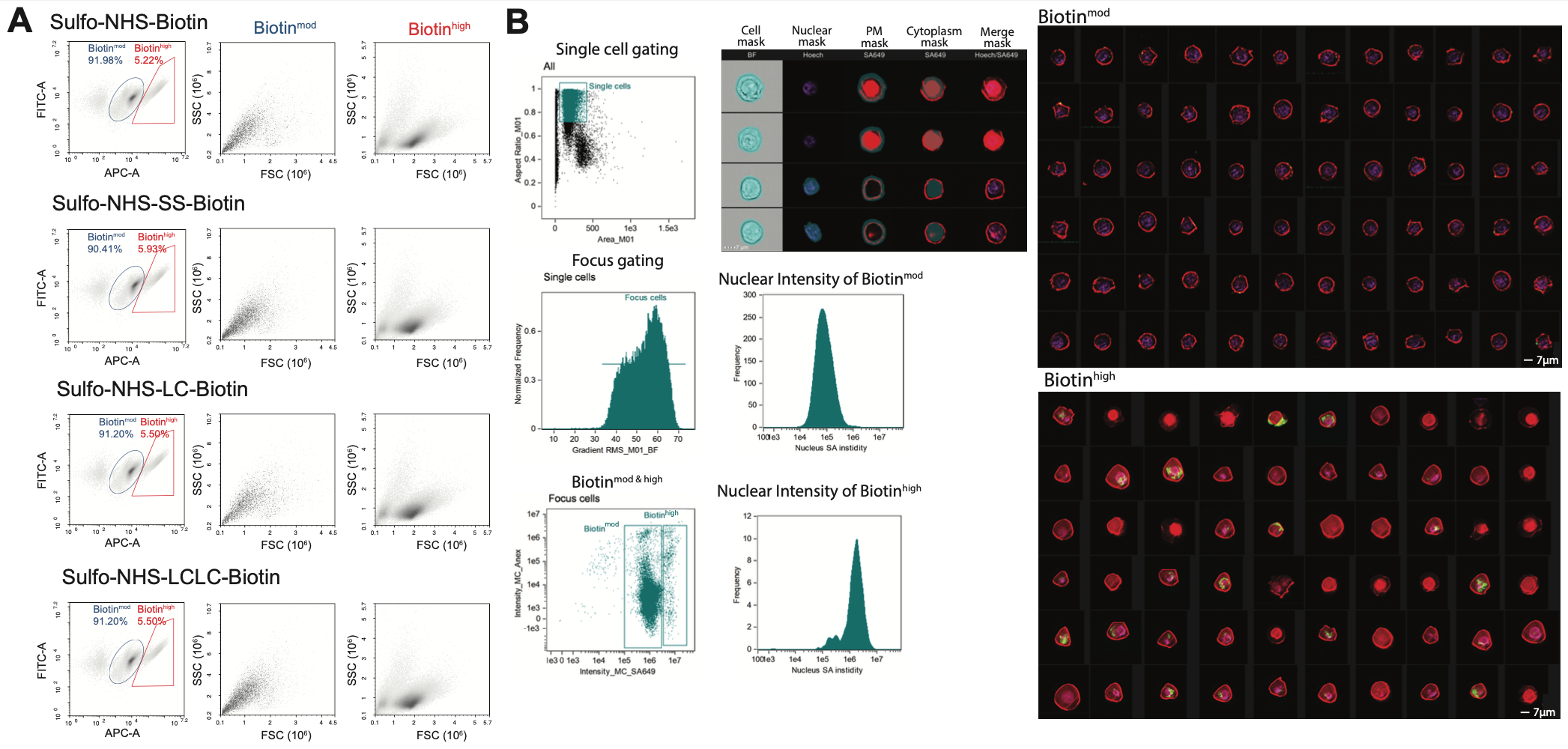
**

**Fig. S1. Evaluation of intracellular contamination by commonly used cell-impermeable biotinylation reagents.**

(A) Scatter plots illustrating two distinct populations biotin^mod^ and biotin^high^ following cell surface labeling with various cell-impermeable biotinylation reagents (left panels). The biotin^high^ population (middle panels) exhibits reduced forward scatter (FSC) and increased side scatter (SSC), consistent with compromised membrane integrity and dead cell contamination. (B) Gating strategy for distinguishing biotin^mod^ and biotin^high^ populations and quantifying intracellular biotin distribution. Sulfo-NHS-biotin–labeled cells were stained with Annexin V–FITC, streptavidin-AF649 (SA-649), and DAPI, and 20,000 events per replicate were acquired by ImageStream®X Mark II imaging flow cytometer. Data were compensated using single-stained controls. Single cells were gated using brightfield (BF) area versus aspect ratio, and in-focus cells were selected by BF gradient RMS. A plasma membrane (PM) mask was generated by subtracting an eroded (0.7) BF mask from the original BF mask. Nuclear masks were defined using DAPI intensity to quantify biotin signal at the PM and in the nucleus.

**Fig. S2. Assessment of intracellular labelling during biotinylation of adherent HeLa cells**

(A) Representative confocal images of HeLa cells stained with DAPI (nuclei, blue) and streptavidin-DyLight-649 (biotinylated proteins, red). Images are shown at 0.5X and 1.2X magnification crops for three independent samples, alongside a negative control that was processed in parallel but without biotinylation (Scale bar=20µm). For quantification, regions with high streptavidin-DyLight-649 signal (“biotin^high^”) were segmented, and the mask was inverted to measure mean fluorescence intensity in the remaining (biotin^mod^) cellular area. Intensities from multiple cells are plotted as a scatter plot. (B) Quantification of biotin^high^ structures across 40 randomly acquired fields. Total cell number per field (DAPI-positive nuclei; blue bars) and the number of biotin^high^ objects (red bars) were manually counted in FIJI. The grouped histogram displays the distribution of total cells and biotin-high spots across fields (top). Scatter plot showing the percentage of biotin^high^ spots per field (bottom). ****p < 0.001 by unpaired two-tailed t-test with Welch’s correction.

**Fig. S3**

**
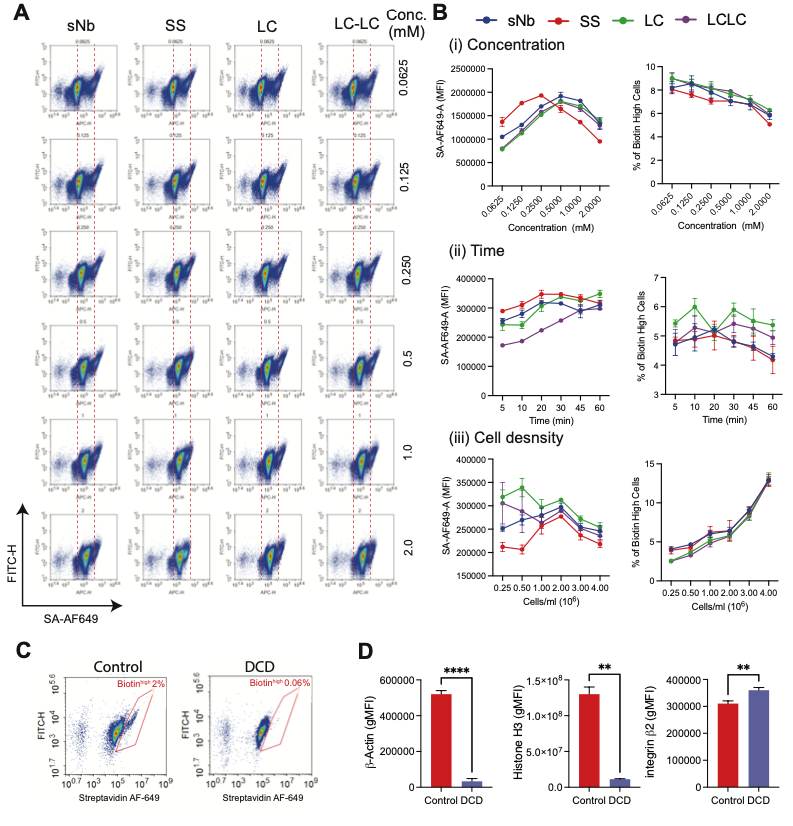
**

**Fig. S3. Optimization of biotinylation conditions to minimize intracellular contamination.**

(A) Representative scatter plots showing the effect of various biotinylation reagents on biotin^mod^ and biotin^high^ populations. Surface labeling was performed with sulfo-NHS-biotin (sNb), sulfo-NHS-SS-biotin (SS), sulfo-NHS-LC-biotin (LC), and sulfo-NHS-LCLC-biotin (LC-LC) as described. (B) Line graphs summarizing the effects of biotinylation reagent concentration, incubation time, and cell density on intracellular biotin signal (left panel) and the proportion of biotin^high^ cells (right panel). For dose-response experiments, cells were labeled with increasing concentrations of each reagent. For time-course experiments, cells were incubated with 0.5 mM of each reagent for the indicated durations. To assess cell density effects, varying numbers of cells were labeled with 0.5 mM sulfo-NHS-biotin reagent for 20 minutes. Labeled cells were stained with streptavidin-AF649 and analyzed in triplicate by flow cytometry.

(C) Representative scatter plots showing CD4⁺ T cells expanded with IL-7 and IL-15, with or without Annexin V–based dead cell depletion. Human CD4⁺ T cells were cultured in the presence of 5nM IL-7 and IL-15 for 14 days, then surface-labeled with sulfo-NHS-biotin, stained with streptavidin-AF649, and analyzed by flow cytometry.

(D) Flow cytometric analysis of streptavidin-agarose bead-enriched biotinylated proteins from control and DCD samples. Cells were biotinylated with or without Annexin V-based dead cell depletion, lysed in 8 M urea, and enriched overnight with streptavidin-agarose beads. Enriched beads were stained with β-actin-FITC, Histone H3-PE, and Integrin β2-APC, and analyzed by flow cytometry. **p < 0.01; ***p < 0.001 by unpaired two-tailed t-test with Welch’s correction.

**Fig. S4**

**
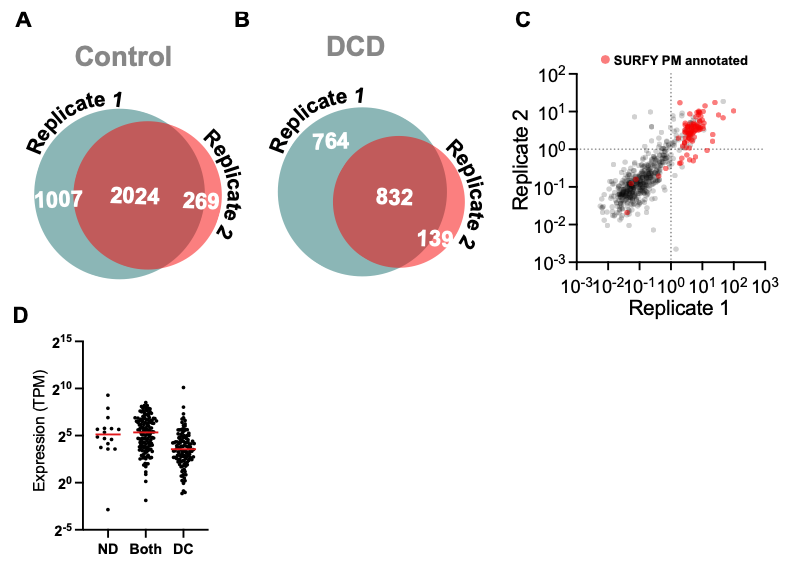
**

**Fig. S3. Reproducibility of dead cell depletion in plasma membrane protein detection.**

(A, B) Venn diagrams showing the overlap of quantified proteins across biological replicates in Control *(A)* and Annexin V-based dead cell-depleted (DCD, *B*) samples.

(C) Scatter plot depicting the correlation of protein enrichment between DCD and Control samples. Non–plasma membrane (PM) proteins are shown in gray; SURFY-annotated PM proteins are shown in red. (D) Dot plot displaying mRNA expression levels of PM proteins identified exclusively in Control, exclusively in DCD, or shared between both. Jurkat transcriptome data were retrieved from the Human Protein Atlas.

**Supplementary Tables:**

Table S1. Identified and quantified proteins, including unique peptide counts and sequence coverage, from Control and DCD samples.

Table S2: Proteomic and transcriptomic profiling of proteins identified in control and DCD samples.

Table S3: List of reference plasma membrane proteins and Topology annotation of identified proteins in Control and DCD samples.

Table S4: List of PM and Non-PM that are uniquely Identified in Control and DCD samples

Table S5: Annotation of Subcellular distribution and Comparison of reference PM annotation with enrichment score.
